# Challenges in Collecting and Transporting Biological Samples From the Field to the Lab: A Retrospective View

**DOI:** 10.1002/ajhb.70360

**Published:** 2026-11-03

**Authors:** Theodore G. Schurr

**Affiliations:** ^1^ Department of Anthropology University of Pennsylvania Philadelphia Pennsylvania USA

**Keywords:** ancestry, consent, DNA, ethnography, fieldwork, population

## Abstract

In this paper, I take stock of past field research and DNA sample collection conducted in different parts of the world to elucidate how this work was undertaken and the knowledge of human biological diversity it produced. Drawing upon firsthand experiences, I discuss some of the challenges involved in collecting and transporting biological samples from the field to the lab for genetic analysis. Such work can be affected by logistical constraints, sample collection method, participant recruitment practices, collaborative arrangements, and the sociopolitical environments in which fieldwork is being conducted. Over time, sample collection methods have also shifted to less invasive approaches to obtaining DNA samples for analysis, leading to more inclusive participation in genetic studies. In addition, I describe some of the lessons learned from conducting fieldwork among different communities, using photographs to illustrate these efforts. Overall, the engagement with different communities in these anthropological genetic studies has significantly shaped my perspective on human diversity and population history and enriched my understanding about how people manage their lives in different cultural contexts.

## 1 Introduction

This paper revisits past research efforts to explore two primary issues. The first concerns taking samples for biological anthropology research with a focus on methods of sample acquisition, emphasizing practices for anthropological genetics projects. The second reflects on lessons learned while conducting fieldwork with a focus on developing the context for interpreting data generated from these samples. The paper concludes with a short summary of insights gained from conducting field research in different parts of the world.

## 2 The Research Team

For over 35 years, I have conducted anthropological genetic studies that combine ethnographic fieldwork and archival research with laboratory‐based DNA analysis. My early work at Emory University focused on the peopling of Siberia and the Americas and used mitochondrial DNA (mtDNA) and Y‐chromosome (NRY) data to ascertain the origin, number and timing of migrations of ancestral Native Americans to the New World (Schurr et al. 1990, 1999; Torroni et al. 1992, 1994; Torroni, Schurr, et al. 1993; Torroni, Sukernik, et al. 1993; Lell et al. 1997, 2002; Brown et al. 1998; Starikovskaya et al. 1998; Schurr 2004; Schurr and Wallace 2003; Schurr and Sherry 2004). Later, through the Genographic Project, my colleagues and I at Penn extended these efforts through many years of research with Indigenous and native descendant populations from Canada, the Caribbean, Mexico and the United States, enlarging our understanding of the histories of these communities (Zhadanov et al. 2010; Gaieski et al. 2011; Dulik, Owings, et al. 2012; Dulik, Zhadanov, et al. 2012; Schurr et al. 2012; Vilar et al. 2014; Benn Torres et al. 2015, 2019; Gómez, Vilar, et al. 2021; Paulino‐Ramirez et al. 2019, Schurr et al. 2026). Other collaborative studies developed during this same period allowed my lab group to contribute to the growing understanding of the population histories of Australia, Georgia, Kazakhstan, Melanesia, Mongolia, Pakistan, and Turkey, among other places (Huoponen et al. 2001; Friedlaender et al. 2007, 2005; Dulik et al. 2011; Gokcumen et al. 2008, 2011; Nagle et al. 2016; Yardumian et al. 2017; Askapuli et al. 2022; Tariq et al. 2022; Zubair et al. 2020; Schurr et al. 2023). Together, these studies have fostered a global perspective on population diversity and afforded opportunities to conduct fieldwork in many places in the world.

I did not undertake all this research myself. Such projects required collaboration with scholars from different countries and local contacts in the communities in which we worked. It also involved graduate and undergraduate students and postdocs from my lab and those at the national institutions where our collaborators work. Collectively, these scholars conducted ethnographic research, sample collection, and laboratory analyses of DNA samples for our studies, and were given authorship for their efforts. These studies further required significant efforts to obtain research permissions to undertake human genetic research in different countries and to build relationships with academic institutions where our collaborators were based to support them. Thus, when using “we” in the following pages to describe fieldwork in different locations, I am referring to all the people who contributed substantially to the success of these studies.

## 3 Sampling Experiences

### 3.1 Sample Sources

When conducting biological anthropology research, researchers commonly collect samples for biomolecular analysis. Among the samples used for these studies are hair, saliva, mouthwash, venous bloods, blood spots, or buccal swabs from living persons. For our projects, we have used all these sample types as sources of human DNA for sequence analysis. The pros and cons of using different ones are discussed in greater detail below. While there is variability in the quality and quantity of genomic DNA coming from different cellular sources (Lucena‐Aguilar et al. 2016; Trost et al. 2019; Connell et al. 2024; Pearce et al. 2024), over time we have chosen to use methods which are most tractable for field‐based sample collection. In addition, certain studies may require specific sampling strategies to conduct newer kinds of analyses in contemporary populations, e.g., feces for gut microbiome work (e.g., Super et al. 2026).

Regardless of the type, a primary task is transporting these samples from field locations to a home institution to undertake genetic analysis. This is not an insignificant issue since the loss or degradation of samples during this process can greatly impact the study outcome. With sufficient preparation, this process can be usually completed without much difficulty. Yet, for different reasons, this process may not always be easy and straightforward, as the following example attests.

### 3.2 Keep Your Eyes on Your Samples

Back in 1993, I began my dissertation project working among Koryak and Itel'men (Kamchadal) communities living in the Kamchatka Peninsula with the late Rem Sukernik from the Institute of Cytology and Genetics in Novosibirsk (see Schurr et al. 1999). We initiated this project 2 years after the dissolution of the USSR during which time Russia was undergoing tremendous political and economic changes. My experiences there were eye‐opening on numerous levels. I was especially struck by how Russian scientists were forced to work in substandard conditions in the post‐Soviet system.

Our work was also noteworthy because US citizens had not been permitted to work in the Russian Far East since at least the start of the Cold War. A primary reason was that Russian air force and naval installations were located on the southern portion of the Kamchatka Peninsula to protect the country's Pacific coast. For the same reason, most Russians were unable to access the region. In this context, we were fortunate to be granted permission to enter the region to conduct fieldwork there. I remember vividly seeing numerous military jets and anti‐aircraft guns under camouflaged netting as our Aeroflot plane taxied to the terminal of the Yelizovo airport near Petropavlovsk‐Kamchatskiy, the regional capital.

Moreover, this area of Siberia was a major focus of the Jesup North Pacific Expedition led by Franz Boas (Boas 1903). As part of this project, Waldemar Bogoraz and Waldemar Jochelson (and their wives Sofia Bogoraz and Dina Brodsky, respectively) conducted extensive ethnographic research among Koryaks and Chukchi, the reports about which I read with great attentiveness (Bogoras 1904; Jochelson 1908). It was exciting to work in the same places that they had documented a century before and compare my own observations to those they had recorded there.

Upon completing an intensive two‐week period of fieldwork on the eastern side of the peninsula in the Karaginskiy Raion [District], we began preparing to return home. Carrying an Igloo cooler with hundreds of blood samples inside, Dr. Sukernik and I traveled to the Yelizovo airport, where we underwent security screening. Despite having official documentation from both US institutions and Russian national and regional governments to conduct our project, airport security officers refused to allow me to board the Aeroflot plane with the blood samples, as they feared that I had stolen organs from Russians and would be selling them on the black market.

After a prolonged and fruitless discussion with them, Dr. Sukernik and I went to the director of airport security to explain the nature of the project and indicate the need for me to board the plane with the samples. Upon reviewing our documents and passports, the director went to the boarding area with us and berated his subordinates about the situation. He then ushered me through the security line and onto the plane with only 30 min to spare before the flight took off. It was a very close call.

I recount this story as a case study of what can potentially happen when obtaining biological samples and attempting to bring them back to a US laboratory from international locations. Other researchers have likely had similar kinds of experiences working in countries where infrastructural issues, delayed permissions, political instability, or just bad luck have negatively impacted sample collection and transportation. Ever since that time, I have never taken my eyes off the biological samples that we have collected for DNA analysis and always confirmed that the customs officials understood what was being transported in the shipping containers used for domestic and international travel related to fieldwork (see more below).

### 3.3 People Generally Don't Like Needles

As noted above, various kinds of samples can be used for genetic analysis. What we learned through fieldwork is that people generally don't like needles aside from their use in medical procedures, although this outlook varied from community to community. In some cases, the prospect of donating blood samples through venipuncture disincentivized potential participants in our studies because of the transient pain it causes and the unfamiliarity of the people taking the samples. The collection materials needed for blood draws are also somewhat cumbersome to transport in and out of the field as well as expensive (Vacutainer tubes, needle kits, alcohol swabs, biohazard containers, coolers, artificial ice packs, etc.). Furthermore, when working with venous bloods, one must prepare to manage the biohazards associated with this sample type, including the disposal of needles, syringes, wipes and latex (nitrile) gloves used during sample acquisition.

Despite these limitations and concerns, the inclusion of a good phlebotomist on the research team helped tremendously with taking venous blood samples from study participants. This approach worked effectively for our anthropological genetics research in Turkey (Alakoc et al. 2010; Gokcumen et al. 2011), for which we employed a registered nurse as a phlebotomist for the project (Figure 1). During this study, participants were also checked for diabetes and blood pressure and provided with information about their health status. We followed this general sampling process for our research in Mexico (Gómez, Vilar, et al. 2021; Gómez, Schurr, and Meraz‐Ríos 2021) and worked with nurses for our biomedical studies of mitochondrial diseases in Russia and Ukraine, as having large amounts of genomic DNA was crucial for our genetic screening work (e.g., Zhadanov et al. 2006, 2007).

**FIGURE 1:**
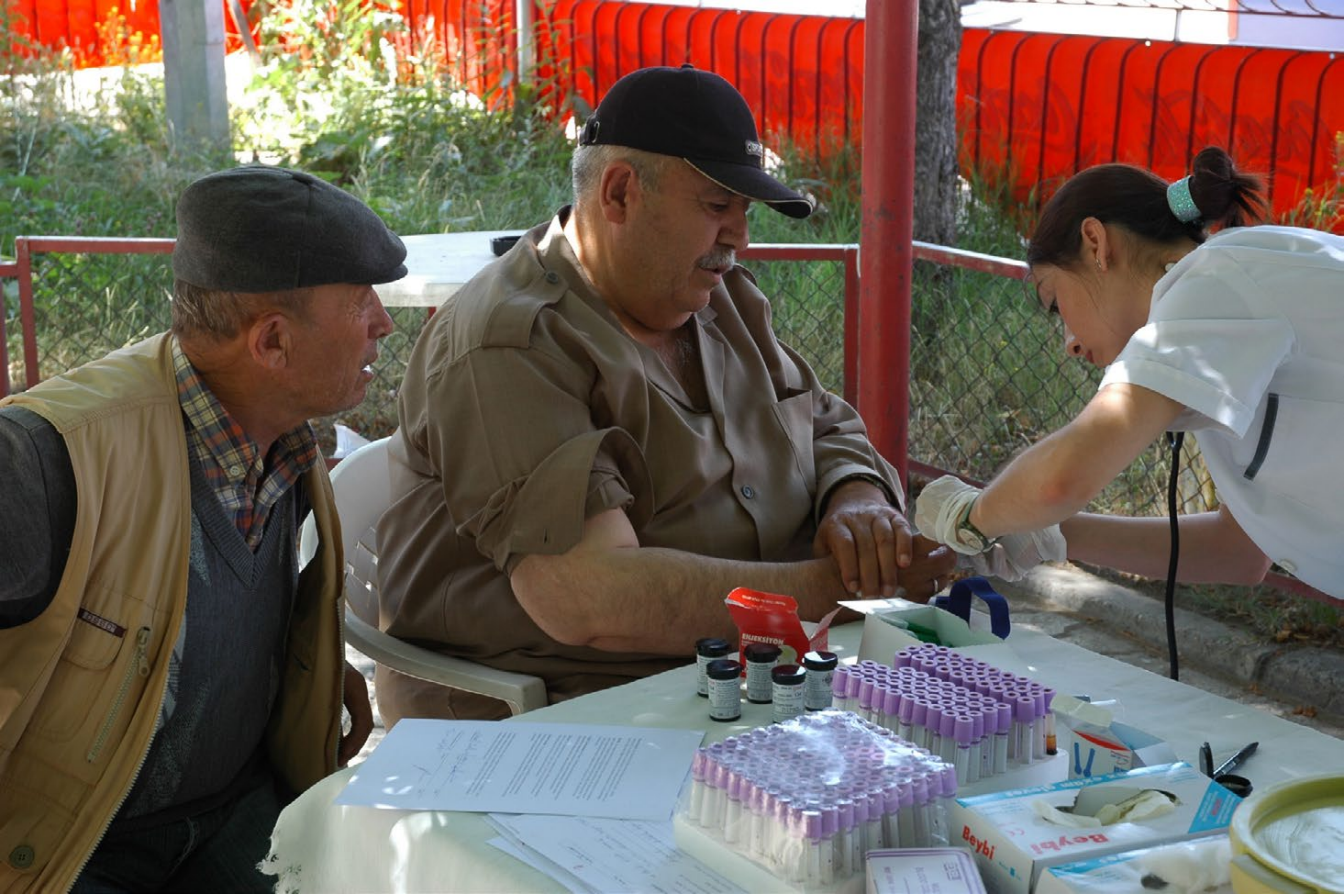
Fatos Yavas, a registered nurse, taking venipuncture blood samples from participants in a village in central Turkey, in 2007. Photo by author.

In general, most anthropological genetic studies today do not employ venipuncture to collect biological samples for analysis. Instead, researchers have shifted to using more non‐invasive methods of sample collection for their studies, including my lab team. Accordingly, over the past 15 years, we have obtained buccal swab samples for nearly all our genetic research in the Americas and Georgia.

### 3.4 Just a Little Swab'll Do Ya

Due to the portability of the kits and the ease of use by study participants, we adopted buccal swabs as our preferred method of DNA sample collection. For most people, swabbing the insides of their mouths (cheeks) is very similar to brushing their teeth or using a toothpick, is relatively quick and painless to complete, and is done under their own volition (Figure 2). The value of collecting buccal swabs has also been enhanced by the availability of DNA sequencing and genotyping methods that can generate significant genetic information from small amounts of DNA with a greater resolution than was possible when I first began conducting anthropological genetics research (Goldfeder et al. 2017; Eren et al. 2022; Lu et al. 2025). We have used this sampling approach for most of our recent studies and enrolled thousands of people in our projects in the process (e.g., Dulik, Owings, et al. 2012; Benn Torres et al. 2015, 2019; Paulino‐Ramirez et al. 2019, Schurr et al. 2026; Schurr et al. 2012, 2023).

**FIGURE 2:**
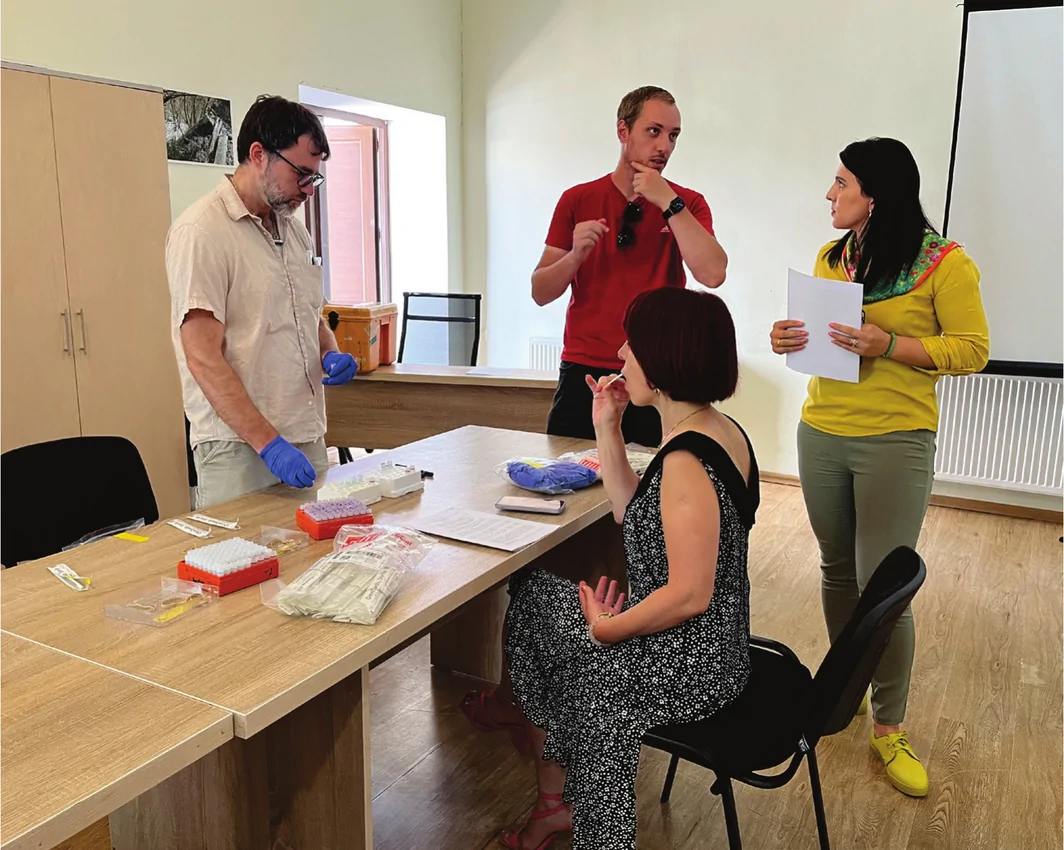
Dr. Aram Yardumian (left) taking buccal swabs from participants in Samtskhe‐Javakheti, Georgia, in 2023. Mikhail Shengelia (middle) is explaining the swabbing process to a participant. Photo by author.

### 3.5 Spit Samples Don't Always Work

For most of our studies, we moved away from using saliva samples for genetic analysis. For one thing, saliva collection requires relatively expensive sampling kits compared to those for buccal swabs. In addition, people vary in their ability to both produce saliva on demand and accurately direct their saliva into collection tubes. In addition, we invariably encountered people who were eating, drinking and smoking and wanted to participate in the study, with these behaviors influencing the amount of saliva they could produce.^1^

Furthermore, we noted gendered differences in public behavior with respect to spitting, whether in a tube or on the ground, or using mouthwash. Saliva collection thus often precluded or reduced the participation of women in our studies (e.g., Puerto Rico; Vilar et al. 2014). As a result, we generally shied away from using saliva kits and focused on sample collection through buccal swabs, although our collaborators in Pakistan have successfully used saliva kits for DNA sample collection and downstream analysis, largely with men (Zubair et al. 2020; Tariq et al. 2022).

### 3.6 Sampling May Happen Anywhere

In my experience, taking biological samples for DNA analysis can happen anywhere, and I mean literally anywhere. We have worked with participants in their homes, in and around churches, and at administrative centers. We also enrolled people at local clinics, schools, tribal community centers, museums, libraries, university conference rooms, and historical societies. In addition, we engaged participants at powwows, in fields outside villages, at old bus stops, and in local outdoor parks, among other locations (Figures 3 and 4). We even enrolled members of police departments at local precincts in Georgia, Puerto Rico and Turkey (Gokcumen et al. 2011; Vilar et al. 2014; Schurr et al. 2023). Thus, almost any location in the field can potentially become a sampling site.

**FIGURE 3:**
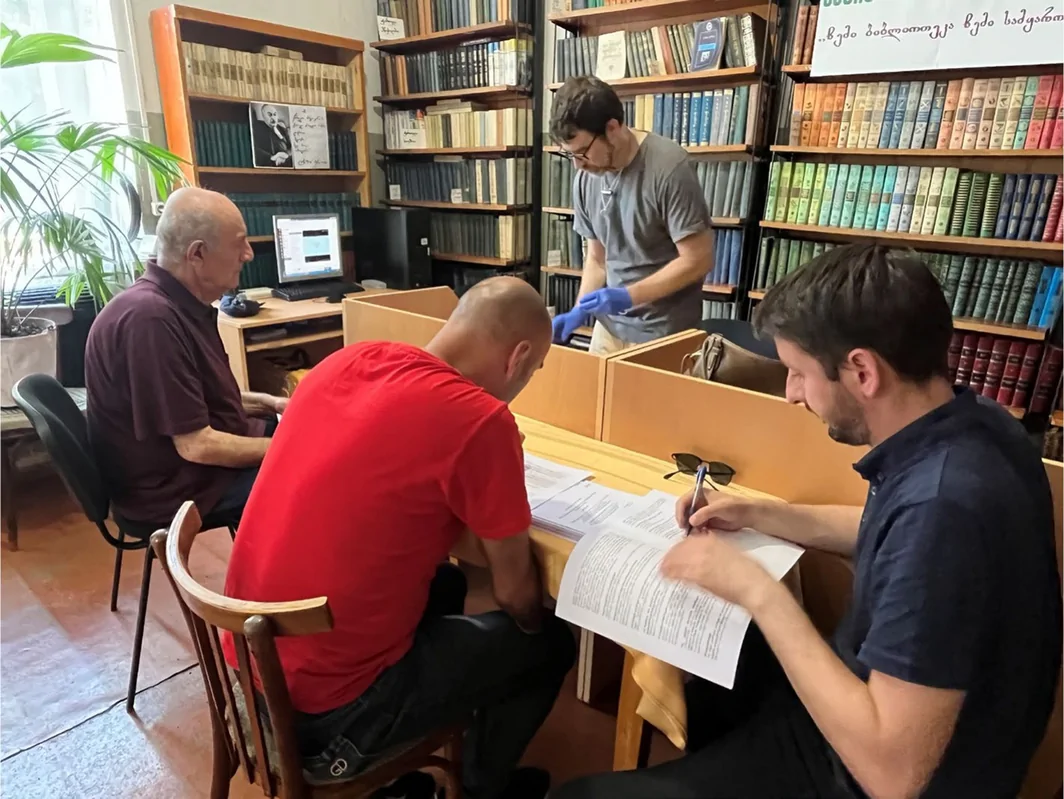
Working with participants in the Rabati Library in Samtskhe‐Javakheti, Georgia, 2023. Dr. Aram Yardumian (upper center) is taking buccal swabs while Levan Jojua (lower right) is consenting participants and collecting genealogical information from them. Photo by author.

**FIGURE 4:**
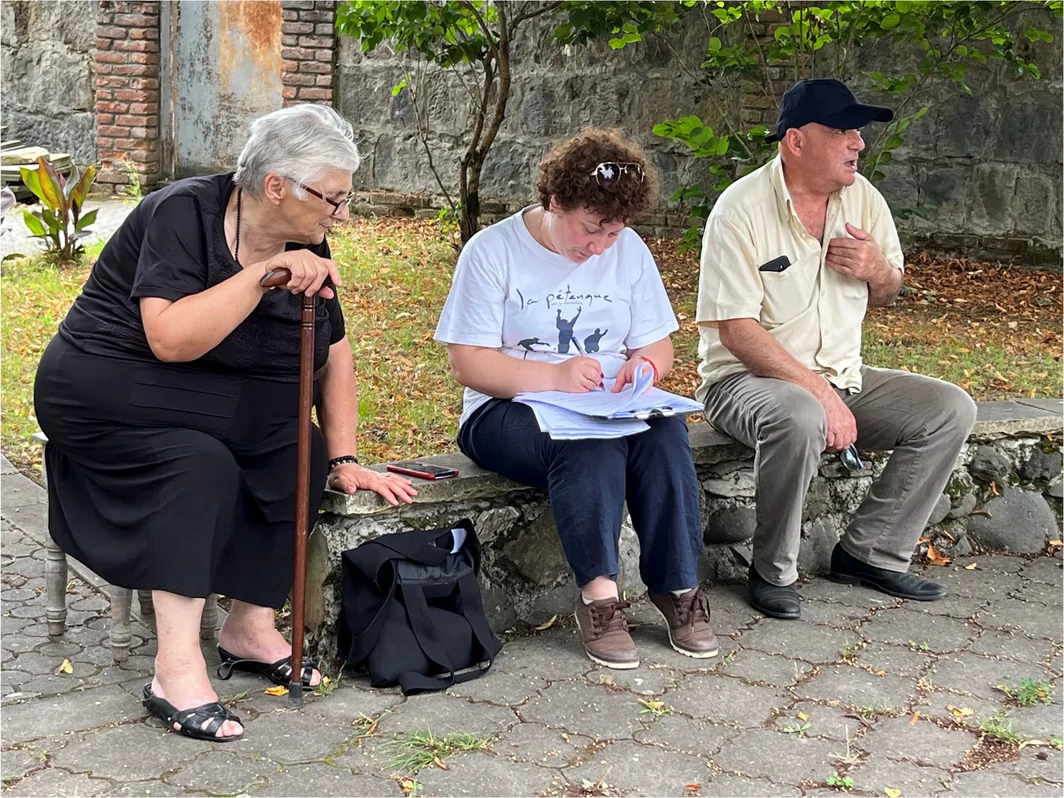
Dr. Shorena Laliashvili (center) interviewing a local historian (Guli Imnaishvil in black dress) in the central park of Shukhuti in Guria, Georgia, 2022. Photo by author.

None of this work happens entirely randomly. We must organize our sampling strategy in advance, coordinate our work with local contacts and collaborators, and prepare for sampling with our research team. These efforts often take many months to plan before undertaking fieldwork and sample collection in a particular location (see Section 3.7).

Once in the field, things may change. We have had to improvise our sampling efforts due to inclement weather, the unavailability of certain spaces, village holidays involving a lot of drinking or a local contact forgetting about our scheduled visit to a specific location, among other reasons.^2^ Sometimes, we encountered unplanned opportunities to takes samples from interested persons. In the end, we basically met people where they lived and worked to enroll them in our studies.

### 3.7 Sampling Logistics

At this juncture, it is important to note that the logistics of our fieldwork varies somewhat from that of traditional ethnographic studies in anthropology. Because we collected biological samples that require processing and analysis in my laboratory at Penn, our ethnographic work in each location was typically undertaken over several weeks. By contrast, traditional ethnographic work may involve 6–18 months of focused engagement within a community. Through repeated visits to specific regions (e.g., Georgia) over many years, however, we gained greater familiarity with the communities in which we work and absorbed important details about their cultural traditions and population history.

Regardless of the duration of fieldwork, arranging the logistics of our projects before we obtained any biological samples for genetic analysis required considerable effort. While the actual time to prepare for fieldwork varied by community, country and location, there were common elements to these expeditions. The most crucial of these—obtaining permission to conduct fieldwork—usually involved a 1–2‐year application process. The process included phone calls and emails with the respective regional or national institutional review boards (IRBs) and often involved in‐person visits to the community and country in which the proposed study was to be conducted. These visits were very important because they provided opportunities for me to present myself to community leaders and for them to evaluate the potential benefits and costs of the project.

As an example, in the Northwest Territories, I visited several First Nations councils in‐person multiple times over 2 years to explain our project. I also had extended conversations with the territorial IRB leadership about the study during that period. These efforts were facilitated by my collaboration with a Canadian archaeologist who had worked in the region for over 20 years and knew the Indigenous leaders of these communities. Yet, it took almost 3 years to obtain the permission needed to conduct anthropological genetics research with these First Nations.

Once receiving official permission for our studies, we began working with our collaborators and local community leaders to organize the sampling process. Using email and telephone calls, these arrangements took anywhere from 2 to 6 months to complete and were contingent on the availability of both collaborators and communities to participate in the study. Through these discussions and those occurring on the ground, we determined the sequence of locations in which to work, promoted the project among community members, secured accommodations for our team during the fieldwork period, and arranged local transportation for the region in which we are working. Furthermore, we usually spent between 1 and 2 months ordering and preparing sampling materials prior to going into the field.

As these experiences demonstrate, arranging field research is complicated and time‐consuming. The initial steps in setting up projects constitute an essential part of “fieldwork” even though no sample and data collection occur during this time. It is therefore crucial to factor the preparation and permission process into the timeline and budgets of funding proposals, which typically cover only costs of sample acquisition and analysis. As a rule, we spent a disproportionate amount of time organizing and preparing for fieldwork compared to undertaking ethnographic work and sample collection in the field.

### 3.8 Refrigerators Can be Hard to Find

During our earlier field expeditions, we were always concerned about having the capacity to keep our biological samples cool. This concern was mainly motivated because we were taking venous blood samples for DNA analysis and did not want them to lyse at ambient temperatures before returning to the United States, where we would fractionate and freeze different blood cell types for downstream analysis. For this reason, we tried to work in local facilities that would allow us to maintain sample integrity with refrigeration. At certain times, we had to improvise to keep our samples cool.

Here, an experience working with another Russian colleague from the Institute of Cytology and Genetics in Novosibirsk, Dr. Ludmila Osipova, remains indelibly etched in mind. During fieldwork in the Altai‐Sayan region of Siberia in 2003, we needed to process whole blood samples at a local clinic. At that time, the electricity worked but was not always consistent in that region of Russia, with sporadic outages occurring. We were also using older centrifuges and preparative equipment that were not internally refrigerated to fractionate blood samples for future use in genetic studies (Figure 5). As a result, I remained extremely nervous about the sample integrity until able to process the blood cell samples for genetic analysis in my lab at Penn. In the end, we were able to obtain high‐quality DNA data from these samples and complete genetic studies of indigenous Siberian and Kazakh populations (Gokcumen et al. 2008; Dulik et al. 2011; Dulik, Zhadanov, et al. 2012).

**FIGURE 5:**
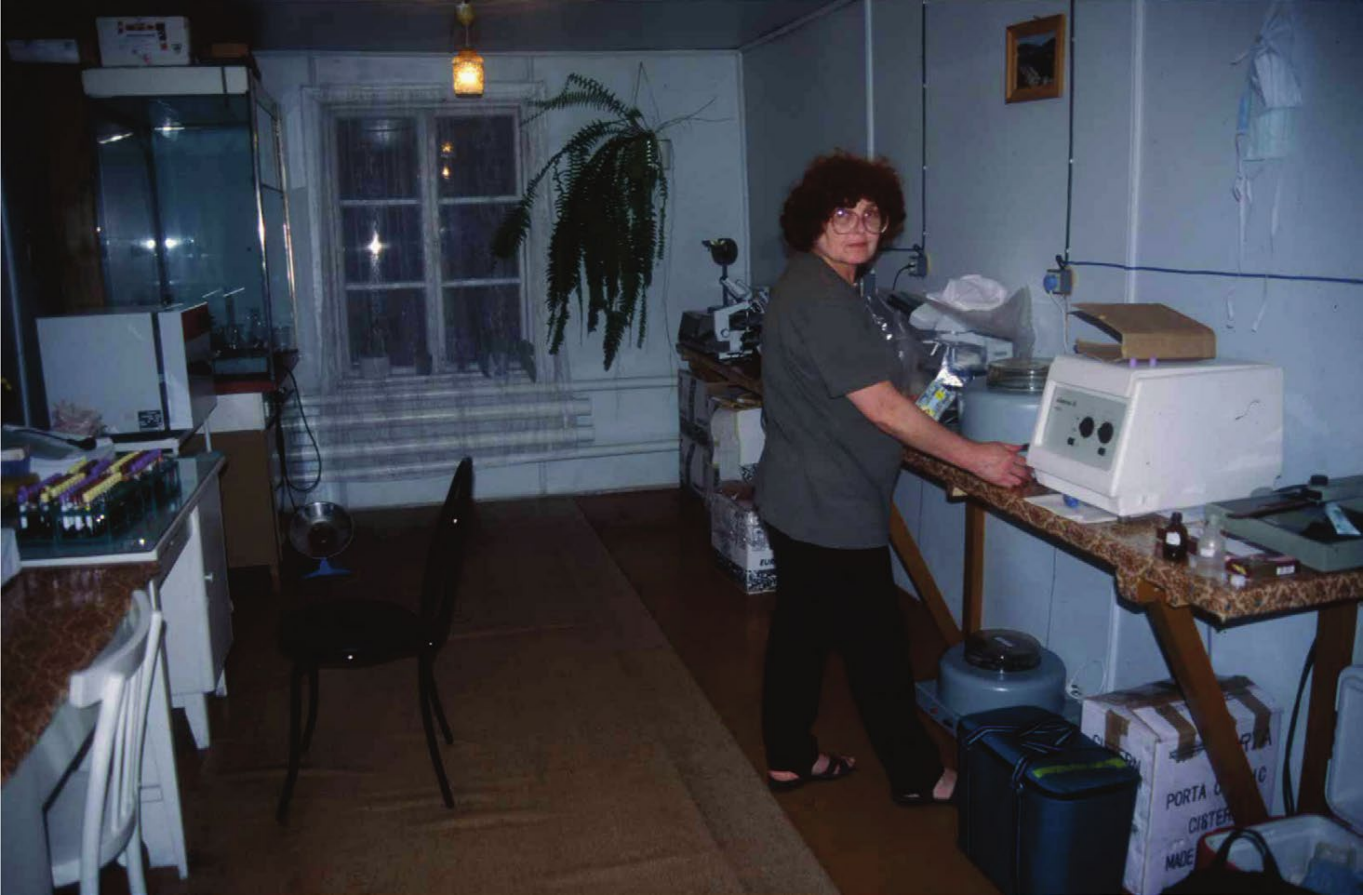
Dr. Ludmila Osipova processing whole blood samples in the Biochemistry Lab at Turochak, Altai Republic, Russia, 2003. Photo by author.

Nowadays refrigeration of biological samples is somewhat less of a concern. Most sample kits contain buffers that allow collected biological samples to be stored at room temperature for extended periods of time. Nevertheless, we have always sought opportunities to refrigerate (4°C) buccal swab samples collected in the field to ensure their integrity and promote the highest yields of genomic DNA with sample extractions upon returning to the lab.

### 3.9 Sampling Requires Teamwork

Perhaps it goes without saying but sampling requires significant teamwork to be successful. The logistical demands of arranging set‐ups to consent, interview and sample participants are not insignificant. As noted above, it takes considerable time to organize these steps in the data and sample collection process at each location, and coordination with local collaborators to notify local community members about the project in advance of our arrival at those places. Sampling of interested persons in the field also often happens in pulses, thus requiring their coordinated movement through each of these steps of the process by team members without undue delays. Despite these challenges, an experienced team, such as that with which I have worked in Georgia for over a decade, can make this work go smoothly (e.g., Yardumian et al. 2017; Schurr et al. 2023) (Figure 6).

**FIGURE 6:**
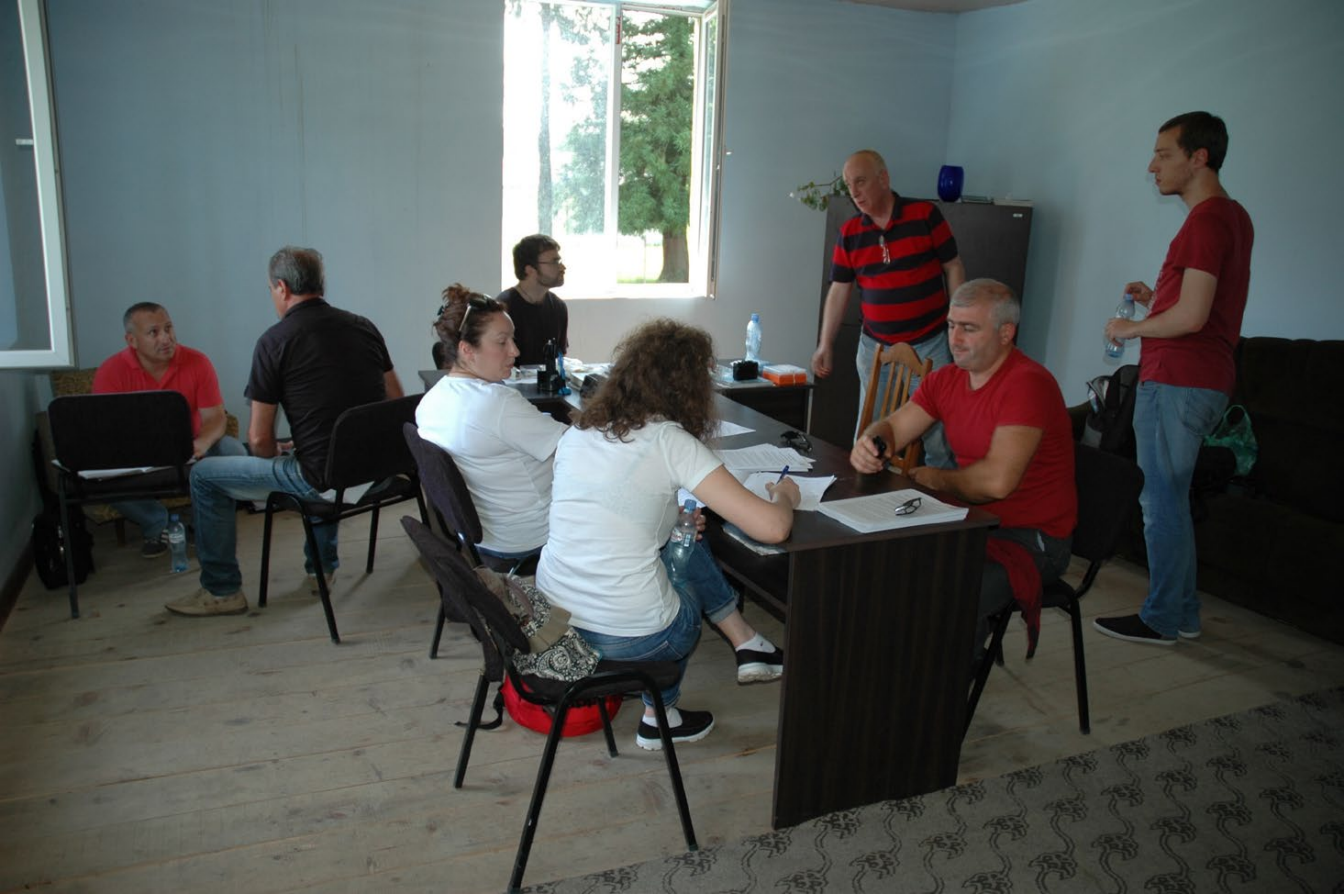
Georgian and American team members enrolling participants from the village of Kurzu in Mingrelia, Georgia, 2016. Photo by author.

With respect to field research in Georgia, our team is comprised of Georgian and American (US) scholars. Besides me, they currently include Dr. Ramaz Shengelia from Tbilisi State Medical University; Drs. Davit Chitanava, Shorena Laliashvili and Lia Bitadze and Mr. Levan Jojua from the Ivane Javakhishvili Institute of History and Ethnology; Ms. Eka Machitidze from the Mikhail Shengelia Museum of the Georgia History of Medicine; and Dr. Aram Yardumian from Bryn Athyn College. Regarding the division of labor for the project, Dr. Shengelia and I organize the fieldwork logistics in Georgia; Dr. Shengelia, Drs. Chitanava and Laliashvili and Mr. Jojua consent participants and obtain genealogical information from them; Dr. Chitanava conducts extended ethnographic interviews with many of the participants; Dr. Yardumian and I obtain buccal swab samples for DNA analysis, with Ms. Machitidze translating the sampling process for participants; both Ms. Machitidze and Dr. Bitadze assist with the interpretation of archival and genealogical information for the project; and I, Ms. Machitidze or occasionally Mikhail Shengelia (son of Ramaz) photograph our field research activities. Once fieldwork has been completed, I coordinate the genetic analysis of the samples at Penn. With most of us serving in these roles for many years, we now work together in a highly coordinated fashion.

This general combination of people is typical for our projects. While the exact number of researchers involved in a specific project has varied, our team usually involves at least 2–3 US scholars, including men and women. We have also collaborated with both male and female PIs in different countries. In our experience, participants did not differ greatly in their behavior toward female versus male team members during the consent, interview and sampling process, although female participants tended to prefer to talk with the women on our team. Furthermore, the local collaborators and community and tribal leaders with whom we worked on these studies included both males and females, with these leadership roles varying by community and the country in which we worked.

### 3.10 Sample Containers Matter a Lot

During the earlier years of our fieldwork, we used Igloo coolers, Styrofoam boxes or other insulated containers made of less robust materials for sample collection and storage in the field. Dissatisfied with these items, my team began using a bright orange case for storing and shipping biological samples and sampling materials. It is made of thick, durable plastic, has padding inside, and is compact enough to be transported on planes and helicopters of all sizes not to mention hauled inside the cars, trucks, minivans and other vehicles used when working in the field. The case is also well‐labeled with my university address and biohazard stickers on the lid to alert people to the potential contents inside and can be locked. In addition, we always place copies of our permission documents in the case, as it will inevitably be opened for inspection by customs officials. Since using this case, we have not experienced any problems with shipping and transporting biological samples for genetic research (as in Kamchatka), whether inside the United States or at international locations (e.g., Canada, Mexico, Georgia).

To elaborate on this point, after retrieving my luggage and exiting the baggage claim area, I am usually stopped by customs officials and asked to open the case and explain what its contents are. During this time, these officials typically photocopy or scan the permissions documents for their records. While these baggage checks take anywhere from 15 to 60 min, the extra time spent affirming the nature of the project and the sampling materials was not wasted. For one thing, it allowed me to demonstrate my official relationship with an academic institution and scholars based in the country where we were working. It also helped to alleviate customs officials' concerns about me bringing illicit substances into the country or trying to avoid paying local taxes on materials with commercial value that I purportedly intended to sell in the country. Sometimes the latter seemed like a larger concern than the transportation of biological samples per se.

## 4 Lessons From Fieldwork

Aside from gaining insights about sampling for genetic analysis through field research, I learned numerous other lessons about conducting anthropological genetics research in different parts of the world (e.g., the Americas, Georgia, Russia, Turkey). In our studies, we have talked with people to learn about their families, local history, and perspectives on their region and community. We have also collected genealogies and oral histories and sometimes checked archival and church records for background information about a location or region. In addition, we often consulted local historians, genealogists, and doctors about their community and the history of the country, as their knowledge greatly assists our understanding patterns of genetic variation in the populations being studied. In fact, most of what fieldwork involved was **not** taking biological samples for DNA analysis but rather developing relationships with the people who were participating in our studies and undertaking ethnographic work in those locations. I reflect on some of these lessons below.

### 4.1 Learn the Local Language

While it again may not have to be stated explicitly, learning the local language in the places one conducts fieldwork, and at least some of the commonly used phrases and salutations, is invaluable for conducting field research. Developing language skills helps one to build relationships with people, shows an interest in their language and culture, and promotes the understanding of these folks and their personal histories. Our questions about specific names or phrases in these languages also often led to illuminating discussions with our local contacts and participants about the history of specific people, places or events. Although learning a new language becomes more difficult as one grows older, in my experience, the effort to gain some competency in a non‐English language has always been a worthwhile endeavor when conducting fieldwork in different parts of the world.

Learning the written forms of some of these languages can sometimes be challenging, though. US scholars typically write and speak in English, which uses a Latin‐derived alphabet for its script. Other written languages may be more difficult to comprehend than the spoken forms because different scripts are used to express them. For me, these languages included Russian (Русский) and Georgian (ქართული), with Mongolian and Kazakh languages using the Cyrillic script to phonetically transcribe them. The Turkish language also now employs a Latin‐based (Türkçe) instead of the Perso‐Arabic script used by the Ottomans (e.g., العثمانيون, “Ottomans”), which is written and read from right‐to‐left.

In the case of handwritten Russian, reading certain Orthodox church records was sometimes complicated. These entries used older forms of specific letters in Cyrillic that had been changed or removed when Peter I introduced his “civil script” in 1708 (Cracraft 2004) or subsequently during the modernization of the script in the 18th through 20th centuries (Comrie et al. 1996; Smith 1998). It was difficult work but yielded small but illuminating details about local village histories and general information about the role that Orthodox priests played in the Russian subjugation of indigenous Siberian peoples (e.g., Slezkine 1994).

For our international research projects, I have been fortunate to work with very capable bi/multi‐lingual collaborators. Their language abilities greatly facilitated the communication of the study's goals and methods to both local administrators and potential participants. When working abroad, we also always translated our informed consent and genealogical forms into the dominant language in a specific location (Turkish in Türkiye, Russian in Russia, Georgian in Georgian, etc.) so that people could read the details of the research study and understand the benefits and risk of participating in it. In some cases, we worked with translators familiar with specific indigenous languages to explain the project to people less familiar with the lingua franca of a region (e.g., Spanish in Mexico). While this step required us to discuss the project in detail with the translators before they communicated the information to local people, it ensured that everyone involved in the consent process clearly understood what participation in the project entailed.

### 4.2 Socializing in the Field

As noted above, an essential part of conducting fieldwork is engagement with members of the communities in which one works. Frequently for us this socializing took the form of eating and drinking together with the people we met. In different places, we received invitations to have tea, consume fresh fruit or sweets or eat local delicacies in the homes of participants. And so, we did this, as it would have been impolite to refuse the hospitality of our hosts. In other locations, after sampling and conducting interviews in a village or town, community members or leaders would invite us to have a meal with them. During these meals, we would often query our hosts about the history, geography and economy of the areas in which they lived, adding to our knowledge of these places. Although varying from location to location, such host‐guest traditions are common in all cultures, with eating together helping to create community among those partaking of the meal (Jones 2008; Dietler and Hayden 2001; Kerner et al. 2015; O'Connor 2015).

For me, some of the most enjoyable social celebrations have occurred in Georgia at the *supras* given in our honor by men of the local communities in which we worked. Literally meaning “tablecloth,” a *supra* is a traditional, celebratory feast with lots of food and wine that features choreographed toasting led by a *tamada* (toastmaster). This ritualized banquet may extend for many hours, giving people time to get to know each other (Manning 2012; Mars and Altman 1987; Dietler and Hayden 2001). At *supras* in recent years, we have been honored by beautifully rendered toasts, *a cappella* performances of traditional Georgian folk songs and the recitation of original poetry honoring Georgian history and traditions. These moments were quite moving and revealed the extent of our hosts' pride in their cultural heritage and pleasure in sharing it with us.

### 4.3 People Love to Talk

Given the innately social dimension of fieldwork, it should come as no surprise to report that, in our experience, people enjoyed talking to foreign researchers who visited their communities. In general, the folks we met were quite open to discussing their family genealogies and the history of the local area, particularly with persons expressing curiosity about these details. Most were also proud of their cultural heritage or history and communicated as much to their interlocutors. In certain locations, the people we encountered had never met Americans^3^ before, leading to various questions about life in the United States, and vice versa.

Overall, we listened and learned from our participants and local contacts, documenting their stories through note taking and audio recordings. Using this approach, we gained considerable knowledge about historical details and events that had not always been recorded in written texts, as well as acquired genealogical details crucial for interpreting our genetic data. Some of these insights have been communicated in published papers describing on our work in different parts of the world (e.g., Zhadanov et al. 2010; Gokcumen et al. 2011; Schurr et al. 2012, 2023; Benn Torres et al. 2015, 2019; Yardumian et al. 2017) (Figures 7 and 8), while others await elaboration in future manuscripts.

**FIGURE 7:**
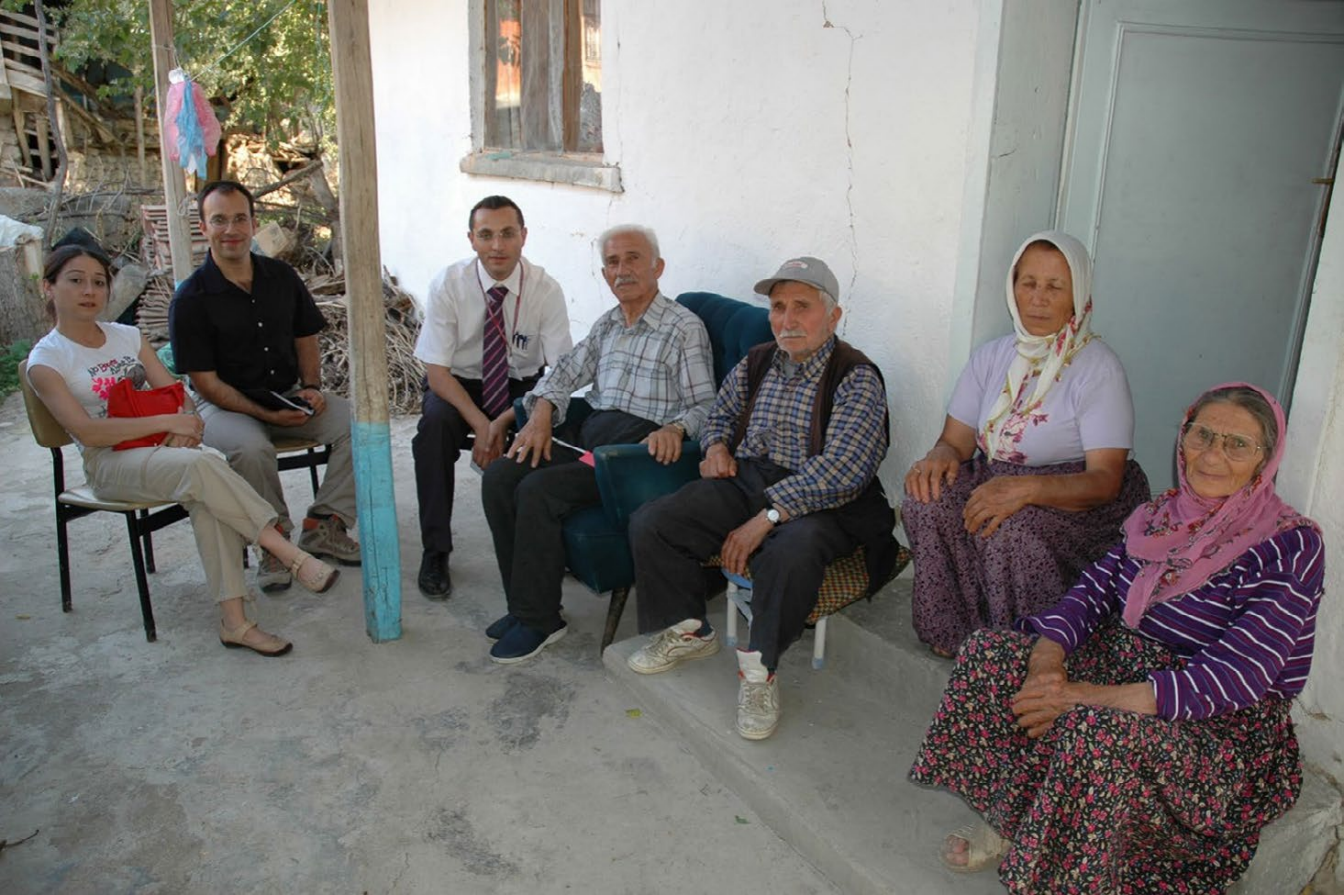
Team members talking with two couples from a village in the Çankiri region of Turkey, 2007. From left‐to‐right, Fatos Yavas, Omer Gokcumen and Dr. Timur Gültekin, with Turkish village residents to their left. Photo by author.

**FIGURE 8:**
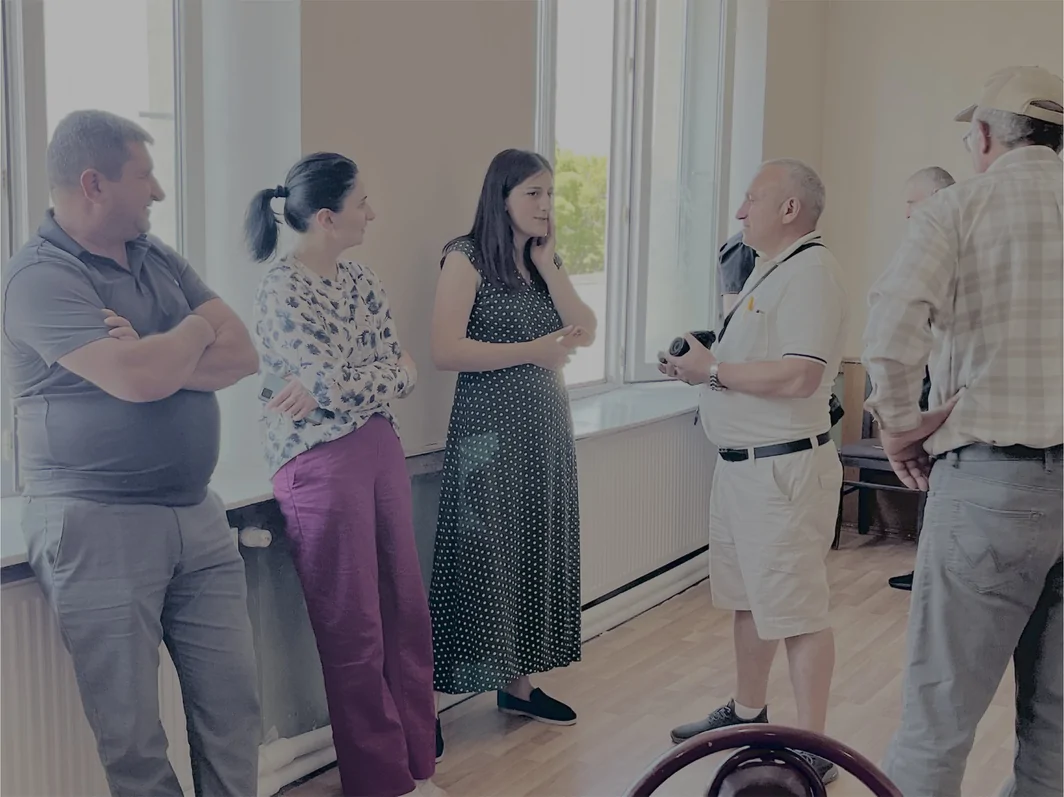
Dr. Davit Chitanava (center right) interviewing participants from the town of Akhalkalaki in Samtskhe‐Javakheti, Georgia, about their genealogies and village history, 2023. Photo by the author.

### 4.4 Extraordinary Experiences

Besides sample collection and participant interviews, fieldwork provided opportunities to experience things that were remarkable, awe‐inspiring or culturally unique. As an example, while working in the village of Biika during a 2003 expedition in the Altai Republic (Russia), I was given the opportunity to feed an East Siberian brown bear cub ( *Ursus arctos collaris* ) (Figure 9). Unfortunately, its mother had been shot by a man in the village and so his family was raising the cub themselves. It was not clear what their ultimate plan for this cub was.

**FIGURE 9:**
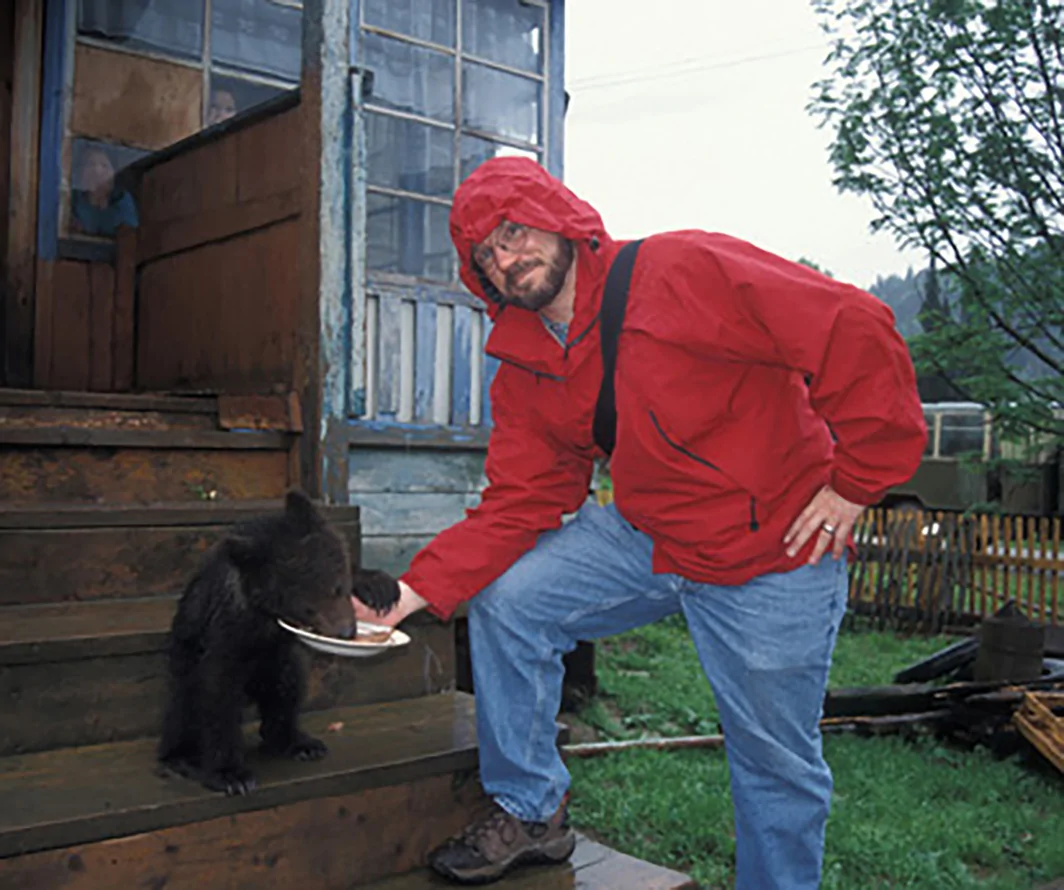
The author feeding an East Siberian brown bear cub in the village of Biika during 2003 fieldwork in the Altai Republic (Russia). Photo by Sergey Zhadanov.

This is just one of the many extraordinary things I experienced while conducting fieldwork in different areas of the world. During a trip to Mexico, I visited the Teotihuacan archaeological site with my colleague, Dr. Rocio Gomez, and we climbed the massive Temple of the Sun to view the vast area it occupied, which attested to its cultural and political significance (Figure 10). I also visited Lake Baikal in south‐central Russia and explored the Bronze and Iron Age burials located along its shores that were the focus of the Baikal Archaeological Project directed by Dr. Andrzej Weber (Mooder et al. 2005, 2006; Weber et al. 2010) (Figure 11). In addition, while exploring possible sampling locations in eastern Turkey north of Lake Van with my student Ömer Gökçumen and Turkish colleagues (Gokcumen et al. 2011), we stopped to photograph Mt. Ararat, which rises majestically out of the landscape (Figure 12). Just before this stop, we were passed by a caravan of people going to a Kurdish wedding in a nearby village, all of whom were clearly in a celebratory mood.

**FIGURE 10:**
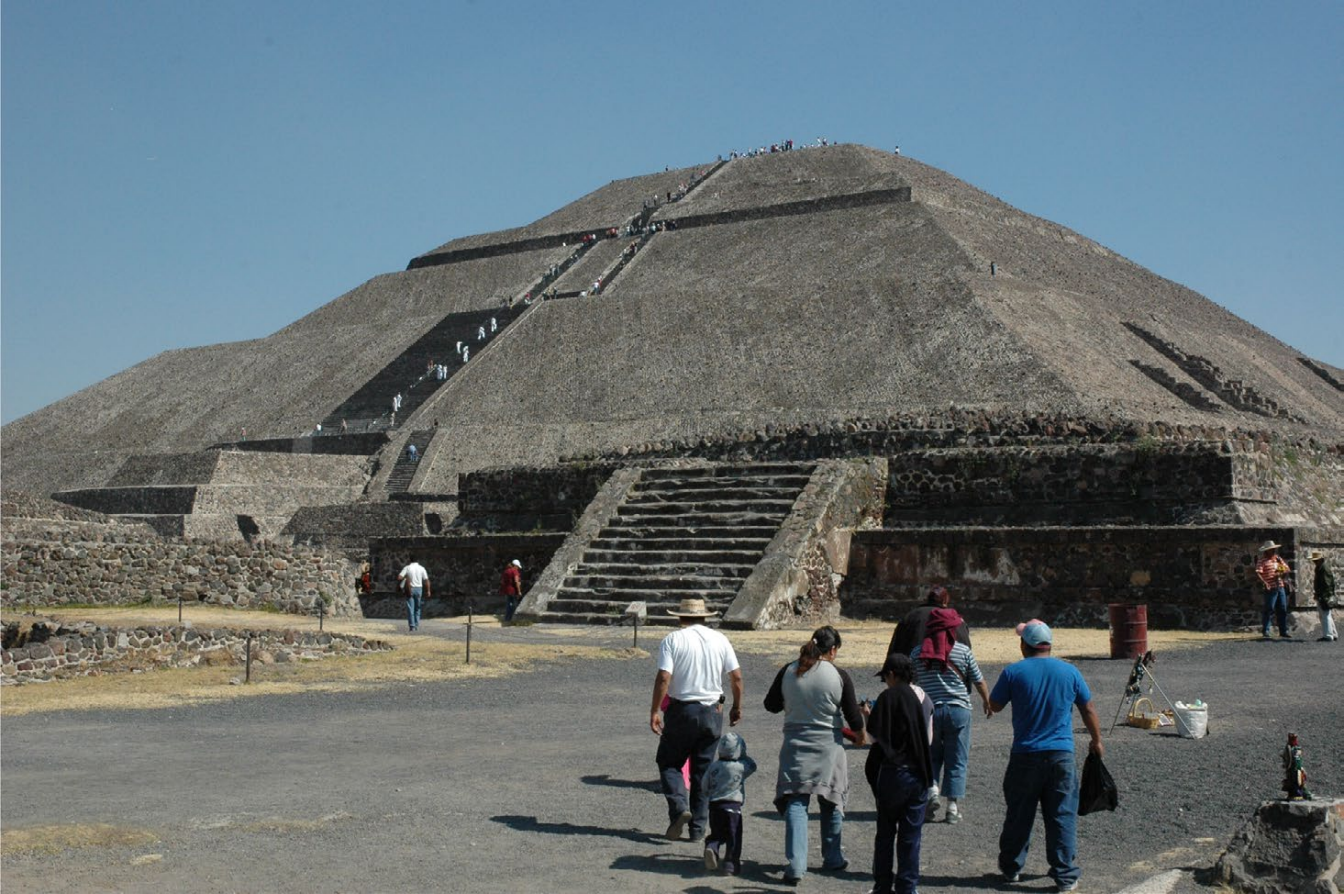
A view of the Temple of the Sun at the Teotihuacan archaeological site in Mexico, 2011. Photo by author.

**FIGURE 11:**
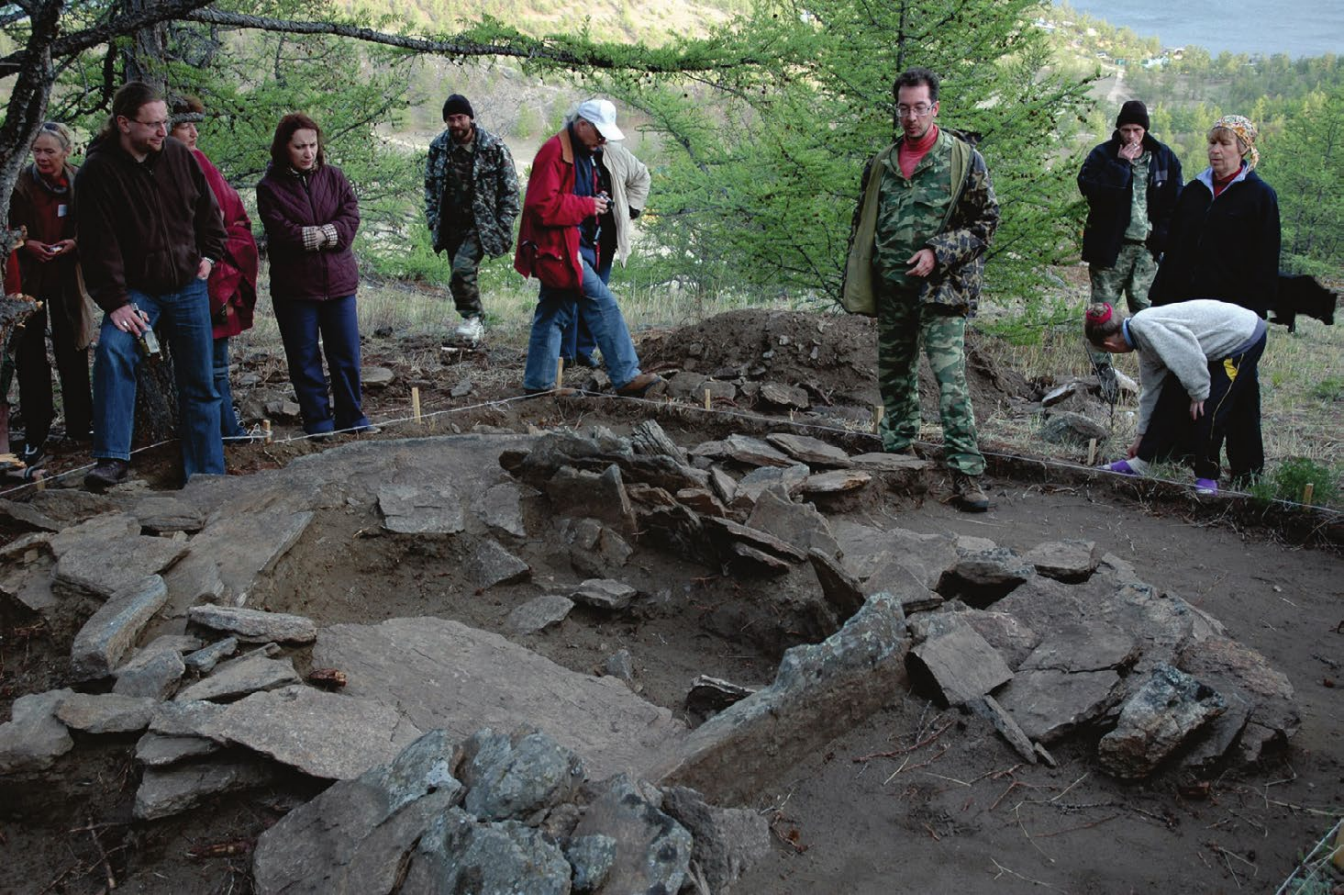
Baikal Project members and conference attendees viewing excavated Iron Age burials along the northern shore of Lake Baikal in south‐central Russia, in 2011. Photo by author.

**FIGURE 12:**
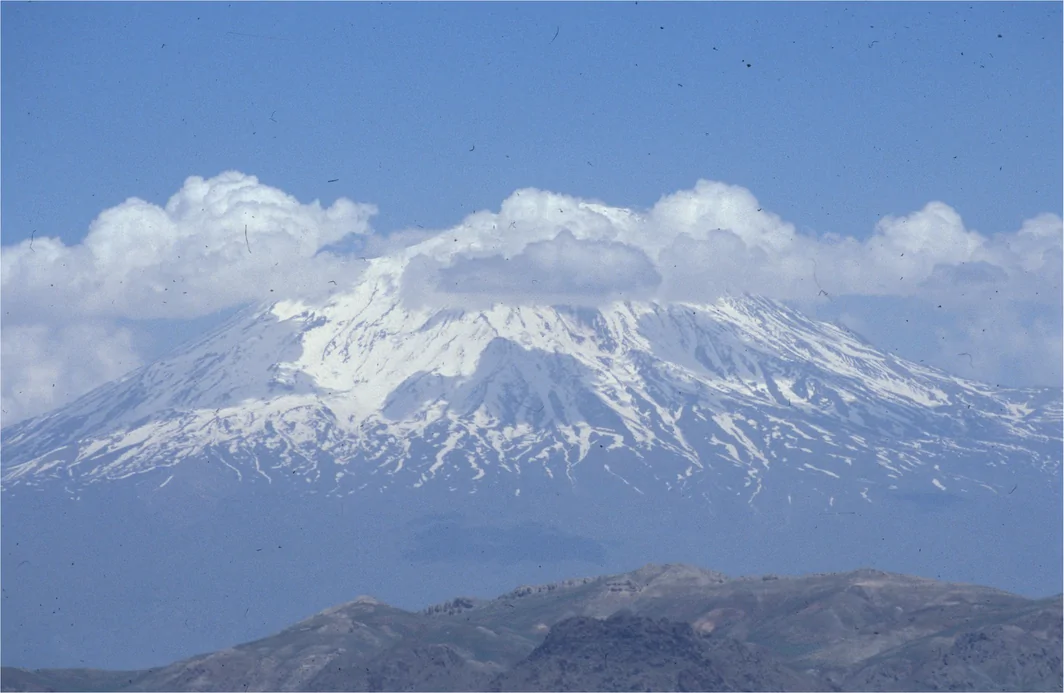
A view of Mount Ararat from Eastern Turkey in 2006. Photo by author.

Besides these experiences, I have had the privilege of engaging in cultural events involving Indigenous communities. I greatly enjoyed watching musk ox wrestling matches at the 2009 Polar Games in Inuvik, Northwest Territories, Canada, along with other traditional skills competitions (Figure 13). I also participated in the installation of newly created totem poles by male and female Haida carvers in Hydaburg, Alaska, in 2012 (Figure 14). Such experiences greatly expanded my appreciation for the diversity of ways that people live in the world and reshaped my understanding of the human history of these regions.

**FIGURE 13:**
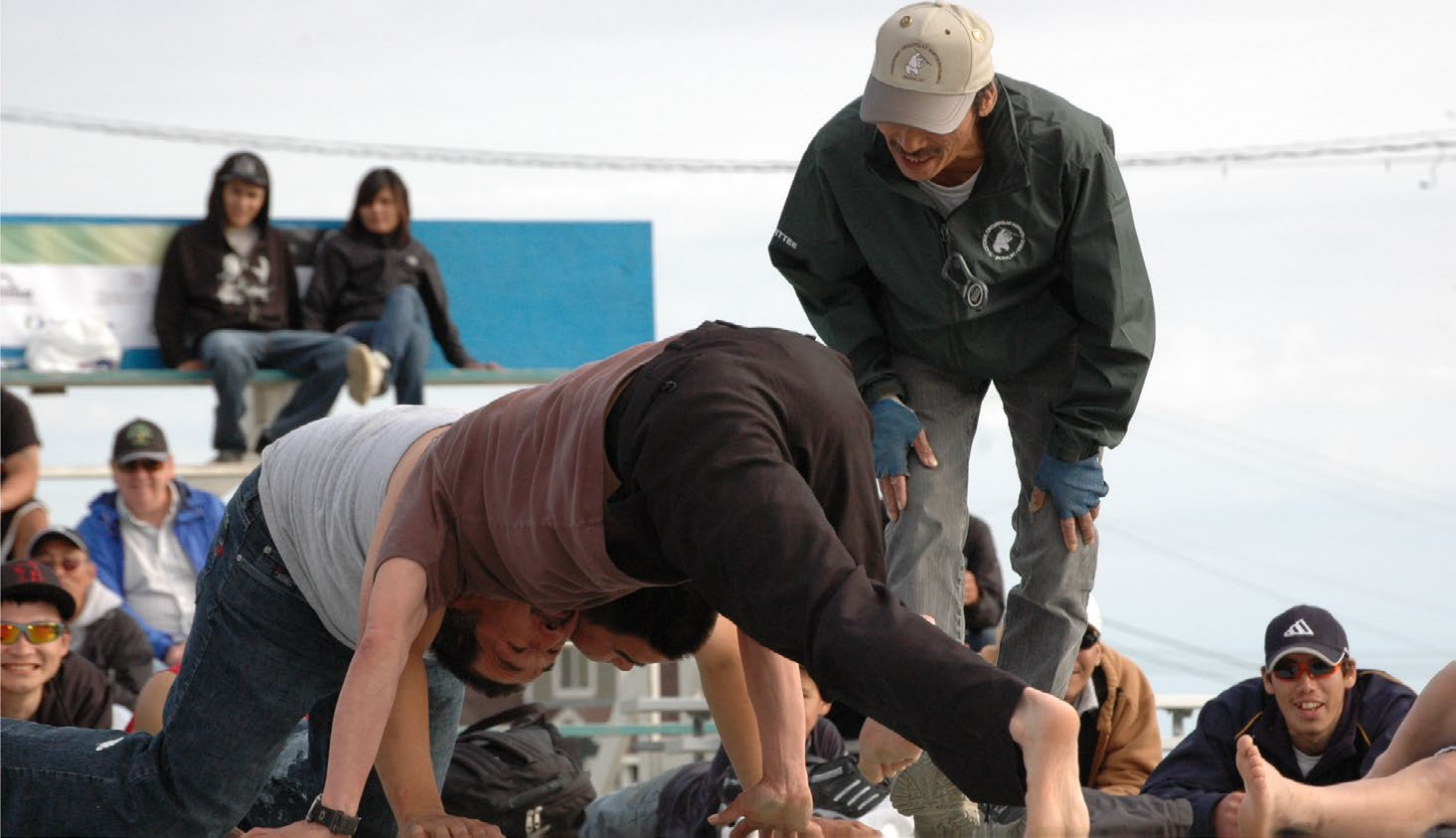
Inuvialuit musk ox wrestling at the 2009 Northern Games held in Inuvik, NWT, Canada. Photo by author.

**FIGURE 14:**
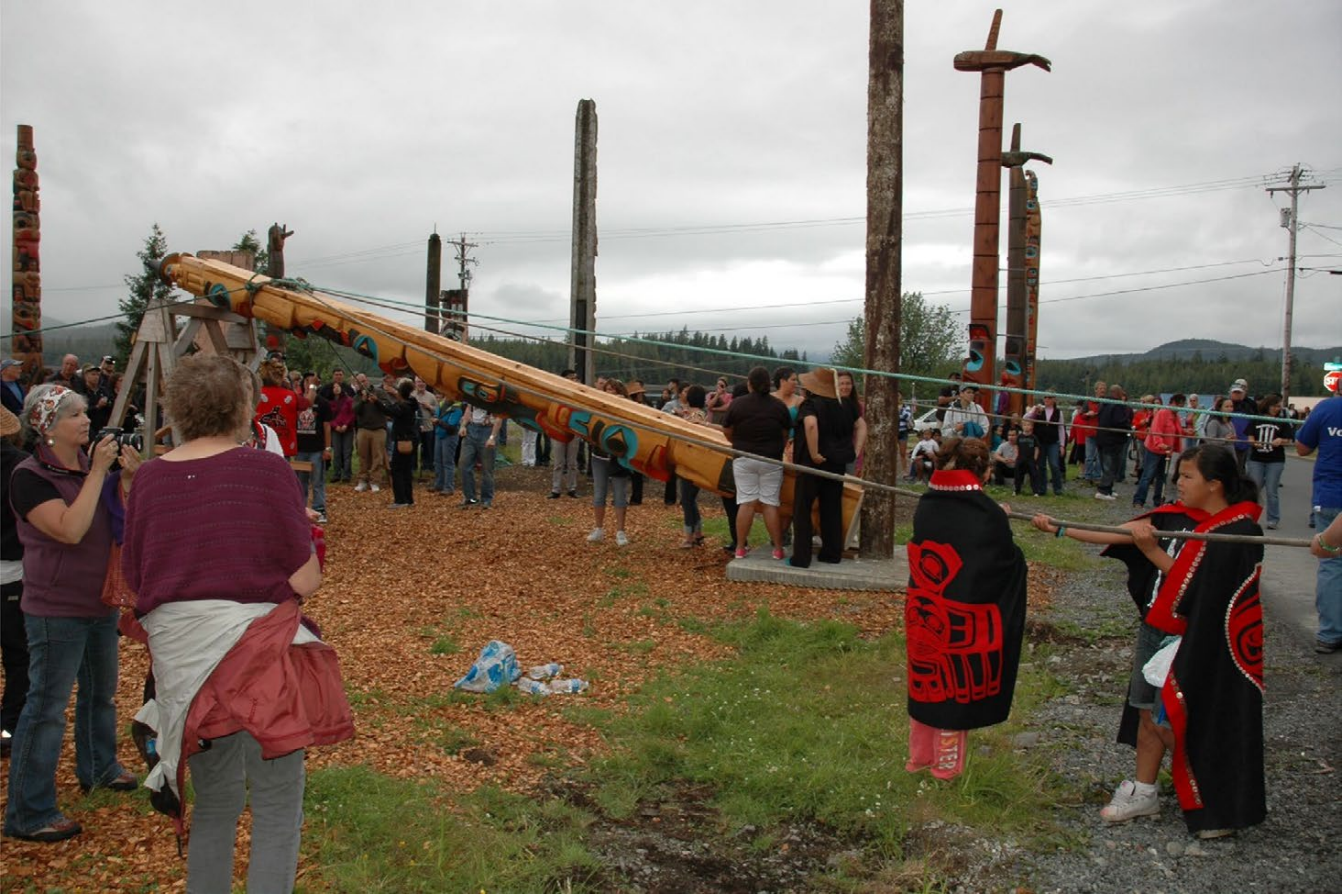
Members of the Hydaburg Haida community erecting a new totem pole created by a female carver in summer 2012. Photo by author.

### 4.5 Finding Your Roots

In every place we have worked, nearly everyone showed a keen interest in learning more about their ancestry and tracing their family roots. While many of our participants lacked a complete grasp of modern genetics, they clearly understood the basics of sexual reproduction, kinship and genealogy, including the idea of parents passing down traits to the next generation. In fact, our participants often joked with each about marrying their siblings or having a different father than their actual biological sire. The results of our genetic research with various communities also usually cohered with published ethnographic evidence for the way social groups reckoned kinship using different descent rules (e.g., Tlingit clan history; Schurr et al. 2012).

While our work has not taught people anything fundamentally new about biological and social reproduction, it has provided opportunities for participants to explore their own genetic ancestry and learn about their distant ancestors in greater depth. To assist them in this process, we provide our participants with detailed summaries of their genetic ancestry results through email and regular mail. For these new insights, participants have expressed their great appreciation to us. Our engagement with them additionally helped us to construct genetic histories more attuned to local population dynamics based on their knowledge.^4^ Moreover, through this research, we have broadened public understanding of science and genetics in communities where discussion of these aspects of human history and diversity have not often taken place.

## 5 Concluding Thoughts

In this paper, I have described some of the challenges of obtaining biological samples of different types for biological anthropology research and the logistics of conducting fieldwork among different communities of people across the world. These observations are based on experiences working in the field and reflect efforts to adjust to the changing contexts in which anthropological and scientific research is undertaken. While not an extensive account, scholars engaged in field and laboratory studies may find these basic insights to be useful for research and teaching purposes.

This paper also makes a modest contribution to the history of biological anthropology. It touches on methodological shifts in the field occurring since the 1990s which have transformed the ways that scholars investigate questions concerning human ancestry, migration history and adaptation. In this regard, I still marvel at the technological advances that have occurred during these few decades, from RFLP analysis and PCR amplification to genomic sequencing and ancient DNA analysis.

On a personal level, these fieldwork experiences have enhanced my ability to conduct anthropological research in different cultural contexts with greater self‐reflexivity and awareness of my positionality as a scholar in the field. They have also opened new opportunities to conduct community based participatory research through which we and our participants have jointly undertaken projects and produced knowledge of mutual interest (e.g., Fleskes et al. 2021). Such experiences have enriched my understanding about how people contend with and adapt to the demands of the socio‐political environments in which they live. Analyzing human genetic variation in populations from different parts of the world has further engendered a deep appreciation for the complicated history of human migration that produced this diversity and the biological and cultural connections we share as members of the same species.

## Author Contributions

**Theodore G. Schurr:** conceptualization, funding acquisition, investigation, methodology, supervision, writing – original draft, writing – review and editing.

## Funding

The author gratefully acknowledges funding support for the projects described herein from the following organizations: the National Science Foundation; the National Institutes of Health; the National Geographic Society; the University of Pennsylvania; the University of Pennsylvania Museum of Archaeology and Anthropology; Ankara University Medical School (Turkey); CINVESTAV‐IPN (Mexico); the Russian Academy of Sciences; and the Social Sciences and Humanities Research Council (Canada).

## Ethics Statement

Protocols for the research projects described in this paper were approved by the Penn IRB #8 and the national, regional and tribal ethics boards in the countries where the projects were conducted. Details about the permission process and research protocols appear in the publications describing these studies. Informed consent was obtained from all participating individuals prior to sample collection and genealogical interviews.

## Conflicts of Interest

The author declares no conflicts of interest.

## Data Availability

No data are being published with this manuscript. Only copyrighted photographs from various research projects are included.

## Endnotes

^1^
This is also a factor when collecting buccal swab samples, with these behaviors generally contributing to lower yields of buccal cells.

^2^
In Russia, helicopter flights between communities were occasionally delayed because the pilot wanted to spend an extra day with a girlfriend in the village where had been working.

^3^
Although I am referring to US citizens here rather than Americans more broadly (i.e., individuals from North, Central and South America), in most places in the world where we have worked people associated the term “American” with persons from the United States.

^4^
There are also implications of our work for the understanding of regional and national histories, i.e., the conceptualization of a “people” or ethnogenesis, which I will not discuss in any detail here.
